# Patient-specific dynamic reference frame for navigation-assisted surgery in mandible: a novel noninvasive technical method

**DOI:** 10.3389/fbioe.2025.1577321

**Published:** 2025-05-13

**Authors:** Jinyang Wu, Lai Jiang, Yingqi Cheng, Wenbin Zhang, Jianfei Zhang, Xiaoyan Gao, Xiaofeng Xu, Shilei Zhang

**Affiliations:** ^1^ Department of Oral and Cranio-maxillofacial Surgery, Shanghai Ninth People‘s Hospital, Shanghai Jiao Tong University School of Medicine, College of Stomatology, Shanghai Jiao Tong University, National Center for Stomatology, National Clinical Research Center for Oral Diseases, Shanghai Key Laboratory of Stomatology, Shanghai, China; ^2^ International Medical Department, Shanghai Ninth People‘s Hospital, Shanghai Jiao Tong University School of Medicine, College of Stomatology, Shanghai Jiao Tong University, National Center for Stomatology, National Clinical Research Center for Oral Diseases, Shanghai Key Laboratory of Stomatology, Shanghai, China

**Keywords:** computer-assisted navigation, 3D printing, dynamic reference frame, patient specific, mandible

## Abstract

**Objectives:**

Computer-assisted navigation has been established as a valuable tool in oral and craniomaxillofacial surgery. However, the steep learning curve associated with mandibular navigation surgery has hindered its widespread adoption. This study introduces a non-invasive, convenient, and accurate navigation method for mandibular surgery and evaluates its clinical effectiveness.

**Methods:**

A modified patient-specific dynamic reference frame (PS-DRF) was designed and fabricated based on the patient’s lower jaw dental cast, integrating navigation technology with 3D printing. During surgery, the PS-DRF was securely affixed to the lower dentition, enabling automatic pair-point registration through fiducial localization via a navigation probe. The surgical procedure was conducted under real-time navigation guidance.

**Results:**

Preoperative registration and intraoperative navigation were successfully achieved in this case. The navigation-guided mandibular surgery was completed without complications. Postoperative superimposition of the simulated virtual model and the actual surgical outcome demonstrated high accuracy, with a deviation of less than 2 mm.

**Conclusion:**

The PS-DRF system offers a convenient, effective, and adaptable approach by integrating navigation technology with 3D printing. This method has the potential to simplify navigation-assisted mandibular surgery and facilitate the broader clinical implementation of computer-assisted navigation in maxillofacial procedures.

## 1 Introduction

Computer-assisted navigation (CAN) is an integrated method that combines computer vision, medical image processing, and real-time localization (Lin et al., 2023). It has been widely applied in oral and maxillofacial surgery, including in areas such as fractures reduction (Pierrefeu et al., 2015), orthognathic surgery (Badiali et al., 2015; Lin et al., 2015), resection and reconstruction of maxillofacial tumors (Palla and Callahan, 2021; Wilkat et al., 2021), and foreign body removal (Gröbe et al., 2009; Gui et al., 2013). The application of CAN in oral and maxillofacial surgery typically includes the following steps. First, preoperative 3D modeling. MRI, CT, and other medical imaging data are used to reconstruct a three-dimensional virtual model, creating a digital anatomical model of the patient, which serves as the key data foundation for the surgical navigation system. Second, preoperative virtual surgical planning and simulation. Using the 3D model, the anatomical features of the surgical area and their relationships with surrounding critical structures are analyzed, establishing a surgical approach and formulating a surgical plan for intraoperative guidance. Third, intraoperative registration and localization. The anatomical data of the patient are aligned with the actual surgical site using a system-compatible spatial locator and probe. Fourth, intraoperative real-time tracking. Using preoperative medical images, spatial localization technologies such as optical or electromagnetic systems (Berger et al., 2015; Brouwer de Koning et al., 2021) are used to measure the spatial position and orientation of the tissues and surgical instruments in the surgical area. This data is then accurately displayed in the surgical navigation system, assisting the surgeon in following the preoperative surgical plan. Compared to traditional surgical methods, navigation systems significantly enhance the visibility of surgical operations and allow for comparison with preoperative planning, helping surgeons adjust their approach to ensure high surgical precision. In confined operative spaces, the system helps avoid damage to critical anatomical structures, making the operation more minimally invasive and safer (Alkhayatt et al., 2024).

Despite the significant advantages of navigation technology in oral and maxillofacial surgery, certain limitations still exist. On one hand, the precision of navigation-assisted surgery is largely dependent on registration accuracy. The selection of registration points primarily involves the localization of titanium screws implanted in maxillofacial bone (Novelli et al., 2014), the placement of surface markers attached on facial skin (Schramm et al., 2009), or the use of anatomical landmarks (Mekuria et al., 2016). Implanting multiple titanium screws in the alveolar bone often results in additional pain for the patient. Surface markers tend to detach easily, which may compromise sterile procedures and increase the risk of unnecessary infections. Furthermore, both of these marker types require the patient to undergo a repeat CT scan to visualize the markers in the imaging data, exposing the patient to secondary radiation. The use of anatomical landmarks, such as dental cusp structures or facial soft tissues, is a commonly applied method. However, it still cannot achieve satisfactory accuracy and stability. Factors such as scanning resolution, the patient’s individual anatomical characteristics, and the surgeon’s subjective judgment and experience can all affect registration accuracy to varying degrees. On the other hand, maxillofacial surgeries typically involve intraoral or minimally invasive small incisions, which require frequent intraoperative adjustments to the patient’s head position and orientation. To achieve synchronous spatial transformations, it is often necessary to make an additional incision in the skull to install a spatial reference frame (Collyer, 2010). This process is cumbersome, has a steep learning curve, increases surgical trauma, and extends operative time.

Furthermore, the mandible, as an independent moving structure in the craniomaxillofacial skeleton, poses challenges for synchronization between the navigation system and CT scans, due to the mobility of the temporomandibular joint (Casap et al., 2008; Bettschart et al., 2012). In multifaceted surgical procedures involving both the upper and lower jaw, such as arthroplasty or double-jaw osteotomy, the use of a navigation system requires intermaxillary fixation (Lübbers et al., 2011), which further limits the surgeon’s field of view and operational flexibility in dynamic clinical settings.

Therefore, there is a significant need for advancements and new technologies to develop a navigation system that is better suited for oral and maxillofacial surgery. Ideally, such a system should enhance accessibility and facilitate clinical adoption by updating compatible hardware that is easy to obtain and implement, while avoiding major alterations to existing surgical workflows. This paper introduces a modified patient-specific dynamic reference frame (PS-DRF) designed to reduce the complexity of navigation-assisted surgical procedures involving the mandible, while improving the minimally invasive nature, stability, and precision of surgery.

## 2 Materials and methods

### 2.1 Materials

A 29-year-old female patient developed severe mandibular deviation within 1 year due to a right-sided condylar osteochondroma. The patient required a precise right condylectomy to achieve complete tumor removal, restore optimal occlusal alignment, and reconstruct a symmetrical lower third of the face. Optical navigation assisted by the PS-DRF was employed to facilitate the surgical procedure. The patient’s authorized consent was signed. This study was approved by the institutional review board and the ethics committees of Shanghai Ninth People’s Hospital, Shanghai Jiao Tong University School of Medicine (SH9H-2021-T66-3).

### 2.2 Methods

#### 2.2.1 Preoperative design and fabrication of the PS-DRF

**FIGURE 1:**
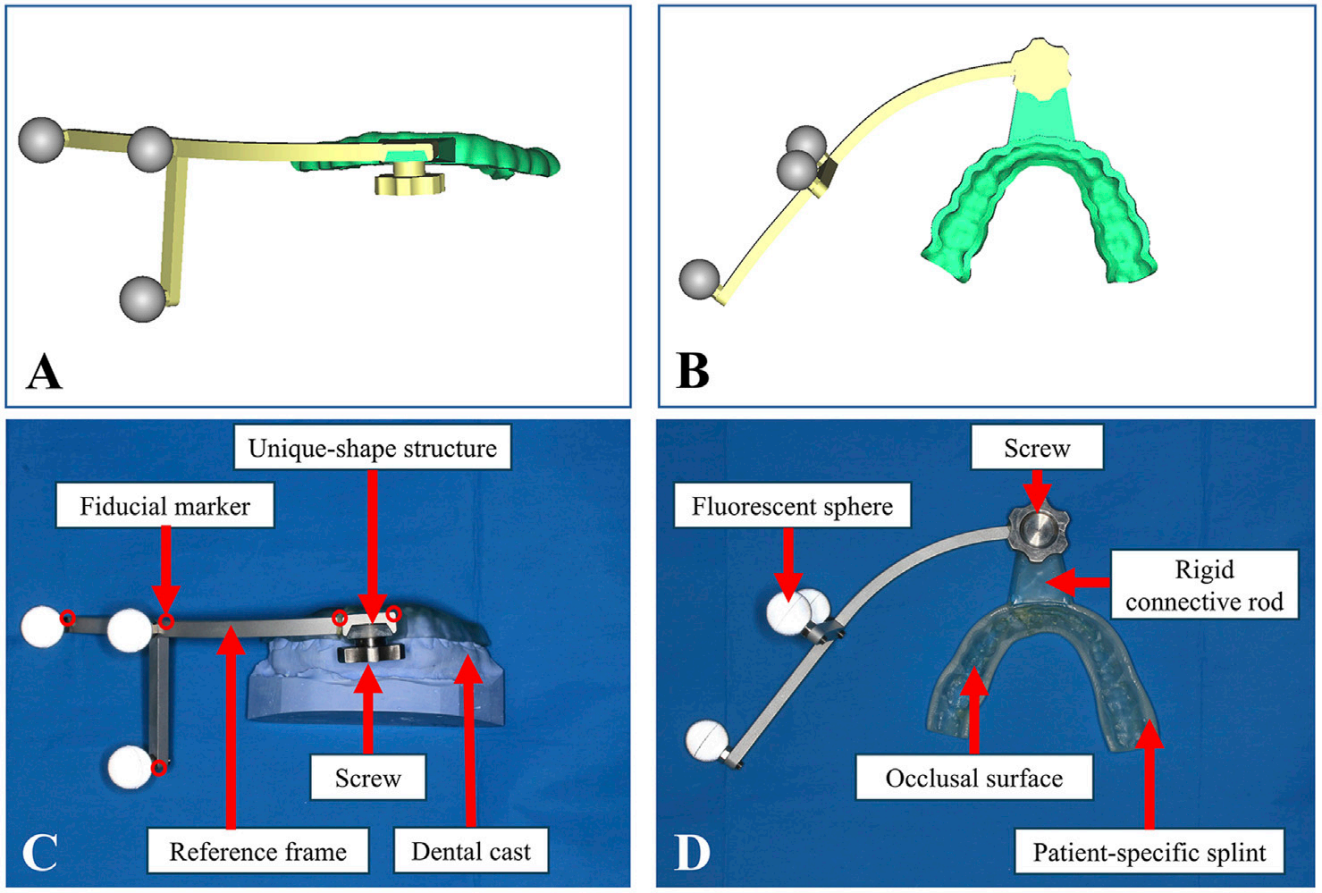
Design and fabrication of the PS-DRF. **(A)** Positive view of virtual PS-DRF. **(B)** Elevation view of virtual PS-DRF. **(C)** Positive view of real PS-DRF showed the structures. **(D)** Elevation view of real PS-DRF showed the structures.

Following the patient’s initial consultation, dental impressions and plaster models were fabricated in the outpatient clinic. The obtained dental casts of upper and lower jaws were scanned using the Activity 880 laser scanner (SmartOptics, Oslo, Norway), generating virtual dental models in STL format. Based on the dental model of lower jaw, a patient-specific occlusal splint was designed in software Geomagic Studio 2013 (Geomagic Inc., Morrisville, NC, United States). The occlusal splint was equipped with a rigid connecting rod. Fiducial markers for registration were incorporated into the splint and rod or set into the reference frame. These markers, represented as 1 mm in diameter and 1 mm in depth conical indentations, enable precise collection of fiducial data during navigation. Due to their rigid structure and distinctive shape, these markers provide greater stability than other maxillofacial anatomical landmarks, such as skin or teeth. The end of the connecting rod contains a unique-shape structure that matches the attachment mechanism of the reference frame. The designed occlusal splint was then fabricated using 3D printing technology via an Objet260 Connex3 3D printer (Stratasys Ltd., MN, United States) whose printing accuracy was up to 200 microns and the MED620 resin material (Stratasys Ltd., MN, United States). A metal reference frame equipped with three infrared-reflective spheres was secured to the occlusal splint via the unique-shape connecting rod and screw at designated positions, as shown in Figure 1. In the virtual environment of Geomagic Studio 2013, the complete assembly of the structure was simulated and integrated with the dental model, followed by export and storage in STL format.

#### 2.2.2 Virtual surgical planning (VSP)

**FIGURE 2:**
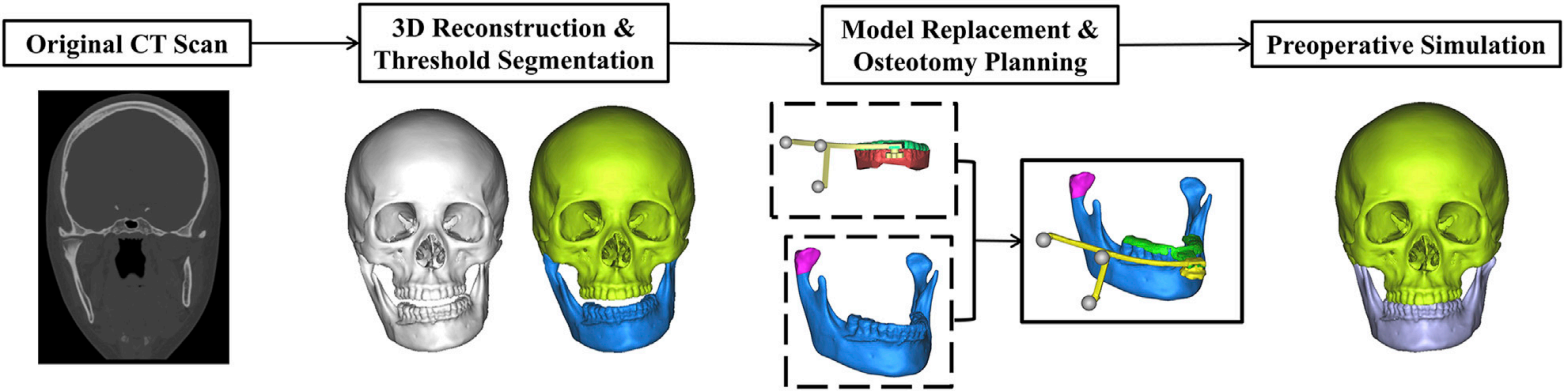
Workflow of virtual surgical planning.

Preoperative CT data was obtained by a 64-slice CT unit (LightSpeed VCT 64-slice Scanner, GE Inc., Fairfield, United States), with slices width and height 512*512 pxl, pixel size 0.488 mm and increment 0.625 mm. Then the CT data from the patient was processed for VSP by experienced surgeons from the Department of Oral and Cranio-Maxillofacial Surgery at Shanghai Ninth People’s Hospital, Shanghai Jiao Tong University School of Medicine. Three-dimensional reconstruction was performed using software ProPlan CMF 3.0 (Materialise, Leuven, Belgium), with structure segmentation based on thresholding and anatomical features, followed by standardized noise reduction and artifact removal. The final STL model of the PS-DRF and dental casts, stored via Geomagic Studio 2013 during the design phase, was then imported into ProPlan CMF 3.0 to enhance the precision of the mandibular dental surface structure. The mandibular dentition reconstructed from CT data was replaced with the dental model of lower jaw via “Create Mandibular Composite Model” tool. The PS-DRF was subsequently registered to its intended position on the mandible via “Alignment tool”. Finally, occlusal alignment complied with the occlusion which was obtained by matching the upper and lower dental casts and recorded by Activity 880 laser scanner. Simulated osteotomy was conducted to remove the tumor and achieve facial symmetry by means of keeping the height of remained ramus same as the health side. If the mandible was still asymmetric, genioplasty or gonioplasty should be simulated. Those ensured that the reconstructive outcome met the preoperative expectations. The detailed workflow is illustrated in Figure 2.

#### 2.2.3 Surgical procedure assisted by optical navigation system

The segmented models from the VSP were exported in STL format and, along with the preoperative CT dataset, were imported into the AccuNavi-A optical navigation system (UEG MEDICAL, Shanghai, China).

**FIGURE 3:**
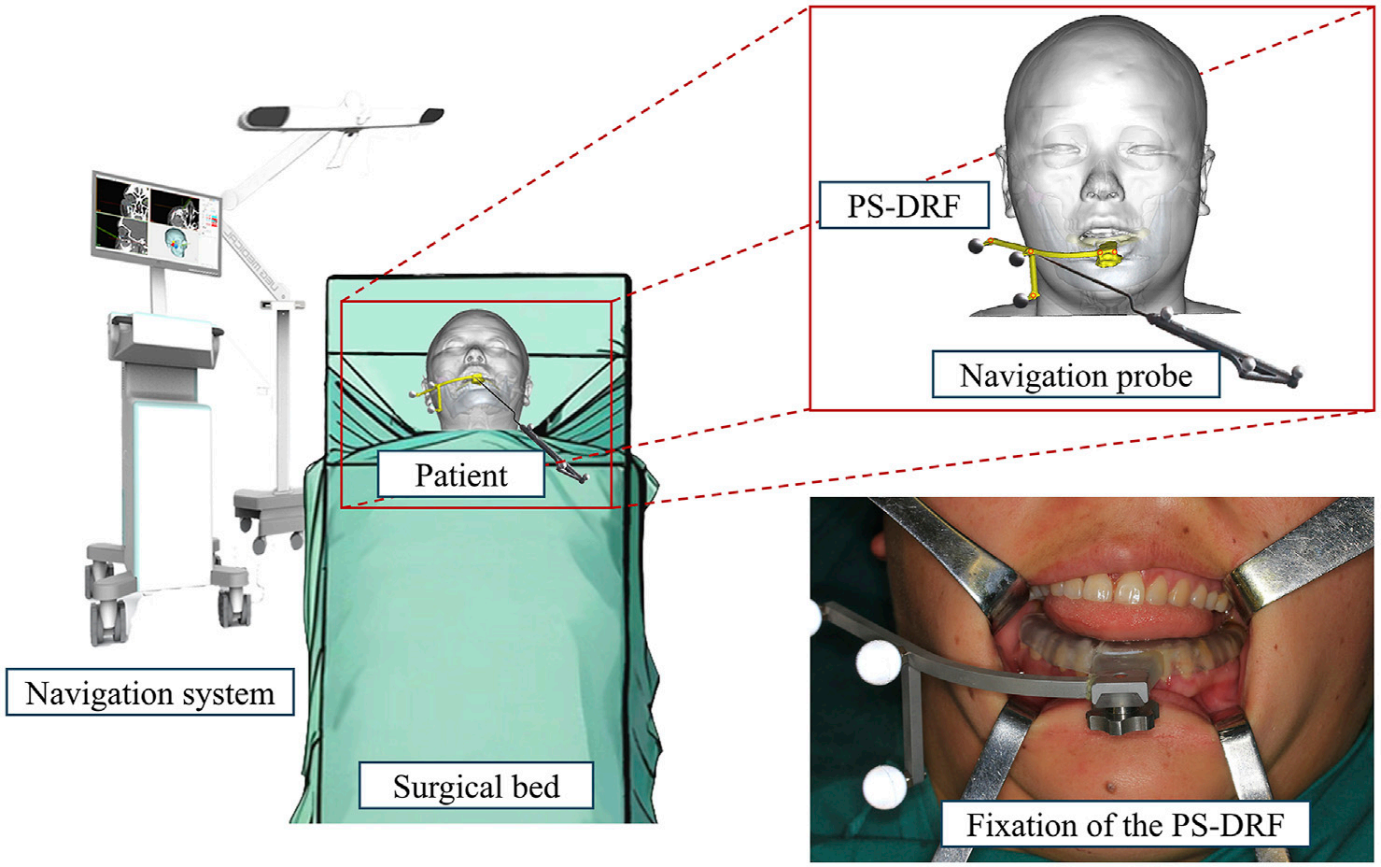
PS- DRF was fixed to the patient’s mandibular dentition.

**FIGURE 4:**
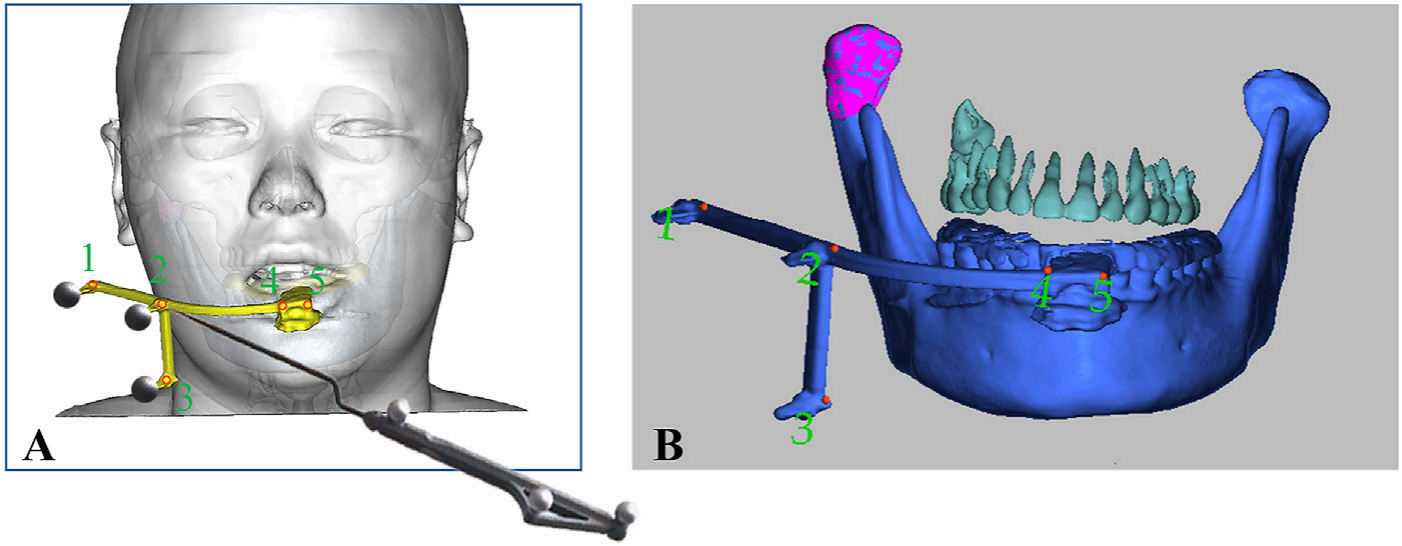
Registration was performed via predesigned fiducial points in the PS- DRF. **(A)** Schema of registration procedure via PS-DRF. **(B)** Screenshot of navigation system showed the registration points in PS-DRF.

After induction of general anesthesia in the operating room, the sterilized and disinfected dynamic reference frame (DRF) was securely fixed to the patient’s mandibular dentition via the patient-specific splint, as illustrated in Figure 3. Therefore, PS-DRF was rigid with the mandible, even mandible was moveable, the navigation system could track the surgical targets in real-time. A passive tracking device emitted infrared light toward the surgical field, and once all reflective spheres were confirmed to be within the detection range, a navigation probe was used to sequentially register five predesigned fiducial points, as shown in Figure 4.

The infrared reflections from the spheres were captured by the tracking device, enabling the spatial localization of each fiducial marker. The system recognized the fixed-array tracker and calculated the spatial position and displacement of the navigation probe relative to the patient, thereby completing the coordinate transformation between the patient’s surgical site and the image-guided navigation space.

**FIGURE 5:**
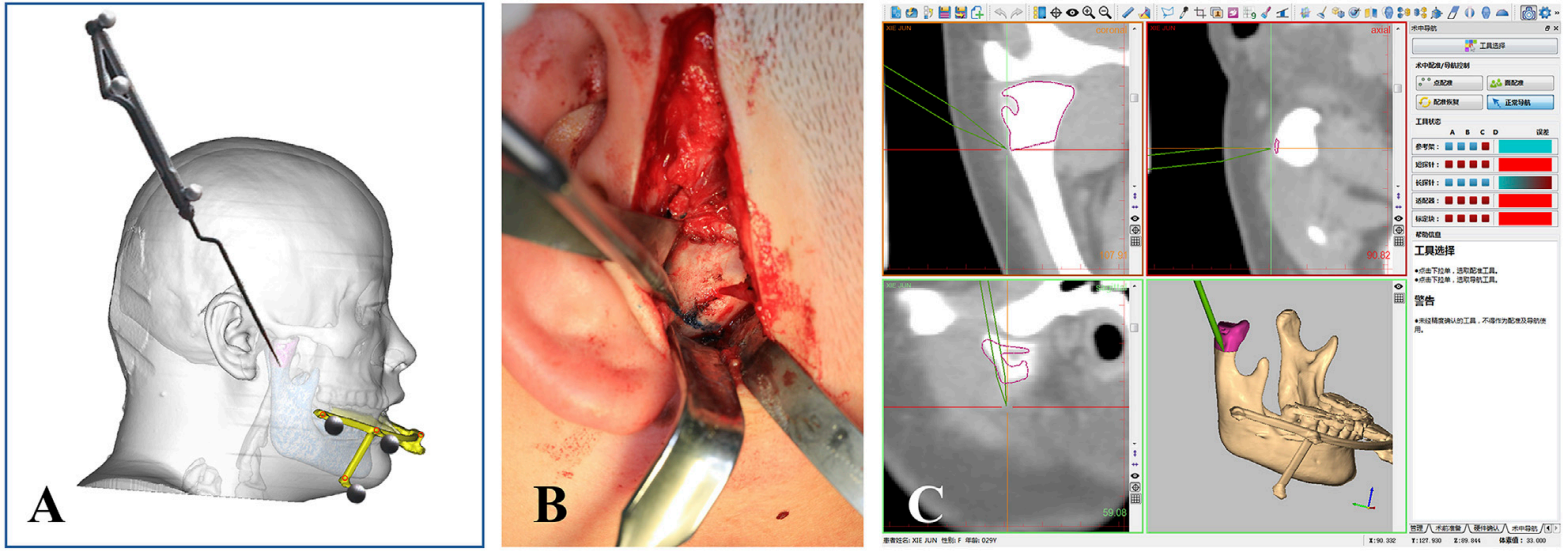
Intraoperative real-time 3D tracking. **(A)** Schema of confirming the osteotomy line via navigation prove. **(B)** Osteotomy line was located by the probe at the surgical area. **(C)** Screenshot of navigation system showed the osteotomy line was being tracked in real-time.

A 3.5 cm incision was designed anterior to the right ear, and dissection was performed to expose the surgical area and reveal the right condylar osteochondroma, as shown in Figure 5B. Next, the osteotomy line was confirmed using the navigation probe, as shown in Figures 5A,C, and condylectomy was performed under navigation guidance. During the osteotomy and tumor removal, the depth of surgical instrument was continuously monitored through the navigation system to avoid damage to critical anatomical structures, such as the cranial base. In the navigation stage, the surgical assistant should keep the PS-DRF fixed rigidly with mandible, or it might cause greater errors or make navigation failed.

## 3 Results and discussion

In this study, the placement and registration of the PS-DRF took approximately 5 min. While there is currently no large-scale systematic study in the literature on the time required for different registration methods in oral and maxillofacial surgery, based on the findings of Badiali et al. (2015), traditional cranial-fixed reference frames add an additional 10–20 min of installation and registration time during surgery. The installation process of the PS-DRF was significantly simplified, effectively reducing the time cost of the registration procedure. Furthermore, traditional optical navigation systems typically require the surgeon to repeatedly adjust the probe position to complete multi-point registration, which can be time-consuming. In contrast, the fixed position design of the PS-DRF’s registration points ensures stability during registration procedure, reducing intraoperative adjustment time, improving overall surgical efficiency, and lowering the technical threshold for the operator.

**FIGURE 6:**
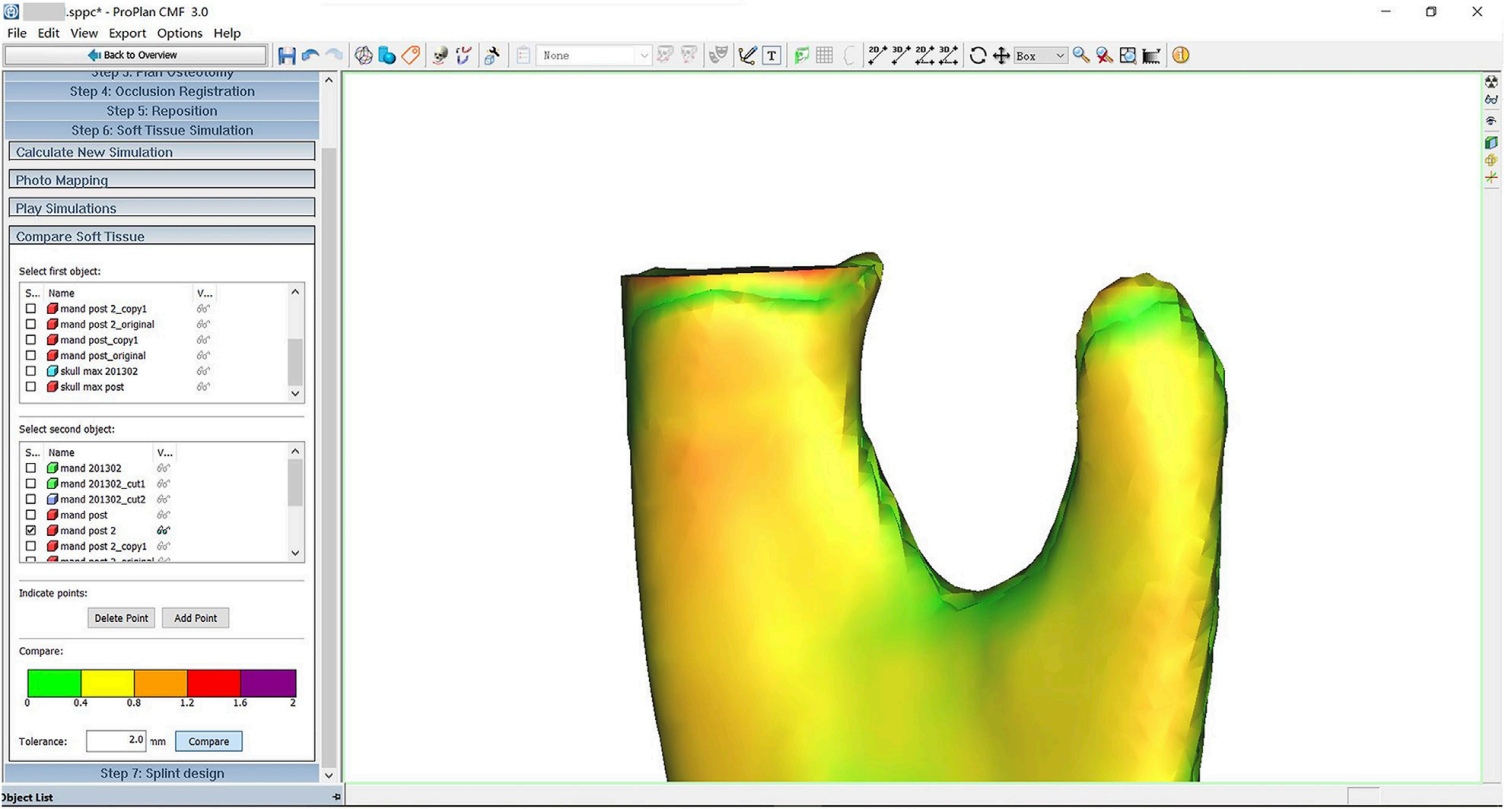
Image fusion showed surgical errors.

Image fusion is one of the key steps in assessing surgical accuracy. During the fitting process, ProPlan CMF 3.0 was used to reconstruct and segment the mandibular models, employing voxel and point cloud computation methods to achieve spatial superimposition and error analysis between the preoperative VSP model and the postoperative actual model. The results of this study showed that the error between the preoperative and postoperative models was less than 2 mm (as shown in Figure 6), confirming the performance of the PS-DRF in surgical navigation could match the clinical demand. Compared to the traditional optical navigation system used by Lin et al. (2015), this method demonstrated comparable accuracy, with the added advantage of clinical safety and stability.

Although this study demonstrated the clinical feasibility and advantages of the PS-DRF, certain limitations still exist. For example, this method may not be applicable in cases where the patient cannot undergo impression taking due to limited mouth opening. Additionally, the dental fixation method may have some limitations in certain special patient populations, such as those with severe dental defects or edentulous patients, and those who cannot cooperate with impression-taking. Future research should focus on designing corresponding solutions for patients with different dental conditions.

In recent years, the application of digital technologies in oral and maxillofacial surgery has significantly advanced the development of precise surgical planning and implementation. Virtual surgical planning (VSP) is one of the key applications of digital technologies. It establishes a patient-specific 3D model using high-resolution medical imaging, enabling comprehensive preoperative assessment and surgical simulation. Many important neurovascular structures in the oral and maxillofacial region, such as the internal maxillary artery, pterygoid plexus, facial nerve, and trigeminal nerve, pass through the soft and hard tissues in deep space. Surgeons typically take great care to avoid injuries caused by these anatomical structures during the operation procedure. Additionally, the surgical approach must consider preserving the function of the oral and maxillofacial system and maintaining facial aesthetics postoperatively, with minimally invasive, precise operations being particularly challenging. Therefore, the precise implementation of VSP relies on the use of subsequent 3D-printed guides or surgical navigation systems. The PS-DRF proposed in this paper integrates VSP, computer-assisted navigation, and 3D printing technology, demonstrating significant clinical value and promising application prospects based on clinical applications.

Traditional surgical guides are typically designed and printed based on preoperative CT imaging data using CAD/CAM technology. The designer can perform repeated simulations and comparisons on the reconstructed rigid 3D model to ensure that the guide fits the anatomical structures of the surgical area and is aligned with the surgical procedure and the patient’s specific needs. Extensive clinical applications have demonstrated that 3D-printed surgical guides significantly enhance the intuitiveness and stability of the operation, thereby greatly reducing the difficulty of surgical procedures. However, due to the variability in patients’ anatomical structures, positional errors may occur during surgery. For example, studies have shown that when dealing with short or thin bone walls in the surgical area, insufficient contact surface area often results in instability (Li et al., 2013). Additionally, the placement of the surgical guide requires adequate space, which often necessitates larger incisions to expose a sufficient surface for guide placement. In cases with narrow surgical access and deep anatomical structures, more time is required for the guide placement. Therefore, the application range of surgical guides in complex maxillofacial surgeries has certain limitations.

The accuracy of computer-assisted navigation has been thoroughly validated, with its core principles lying in comprehensive preoperative planning and spatial registration, followed by the real-time collection and tracking of spatial information to monitor the positions of patient’s anatomical structures and surgical instruments, thus guiding the surgeon to perform precise operations. The registration structures relied upon by traditional navigation systems can be classified into two types: invasive and non-invasive, both of which are highly dependent on the surgeon’s subjective judgment and experience. During long surgical procedures, distraction and physical fatigue present potential risks for human error. Compared to traditional methods, Abbate et al. introduced a method of directly fixing the reference frame to the mandibular ramus to track mandibular movement and assist in segmental mandibular resection and reconstruction (Abbate et al., 2017). Lee et al. proposed a 3D-printed personalized registration framework fixed to the external auditory canal and upper anterior teeth to improve the navigation accuracy in orbital floor reconstruction surgeries (Lee et al., 2019). Yamamoto et al. developed a maxillomandibular support-based reference frame designed to maintain mouth opening during the procedure (Yamamoto et al., 2020), which is used for navigation-assisted curettage of mandibular cyst. These three improvements simplified the registration process without causing additional trauma, making them more acceptable to patients.

The PS-DRF also belongs to the non-invasive registration methods but features a design better suited for oral and maxillofacial surgery. First, registration structures relying on fixation to the external auditory canal perform well when the patient is in the supine position. However, when frequent intraoperative adjustments of head position are required, positional deviations are often unavoidable. In contrast, a registration splint fixed solely to the dental arch overcomes this limitation. Second, the PS-DRF does not come into direct contact with the mandible, preventing potential obstruction of the surgical field, thus offering greater versatility. Additionally, its compact and lightweight design eliminates the need for additional surgical instrument channels. Instead, a new splint can be custom-designed based on the patient’s dental cast, while the reference frame itself can be repeatedly sterilized and reused, optimizing medical resource utilization and cost-effectiveness.

## 4 Conclusion

In summary, based on existing research, the modified PS-DRF offers four key advantages. (1) Stable positioning without additional surgical trauma, while also eliminating the need for a second preoperative CT scan, thereby avoiding redundant radiation exposure. (2) The navigation splint, designed based on specific cusp anatomical structures, moves synchronously with the mandible, ensuring that no significant deviation occurs between the spatial coordinates of the navigation system and the actual surgical space. (3) The conical fiducial marker design facilitates the operators to recognize and locate the markers easily, reduces reliance on both the surgeon’s clinical experience and manual dexterity during registration, and provides high reproducibility. (4) Only a thin occlusal splint needs to be 3D printed for each patient, making the method easily scalable and cost-effective.

In the future, systematic clinical research on the PS-DRF will be a primary focus to further validate its effectiveness and safety. The integration of multimodal data with AI-driven technologies may further optimize the data processing workflow, such as automatic anatomical structure recognition and intelligent planning based on deep learning algorithms. Enhancing the degree of automation will significantly reduce the design and fabrication time of PS-DRF, thereby improving the efficiency of surgical navigation technology in oral and maxillofacial surgery.

## Data Availability

The raw data supporting the conclusions of this article will be made available by the authors, without undue reservation.
